# Supplementary material for: Air Quality, Management Practices and Calf Health in Italian Dairy Cattle Farms
Source: Animals (Basel). 2022 Sep 3;12(17):2286. doi: 10.3390/ani12172286 (PMC9454719; doi:10.3390/ani12172286)
Supplement: Supplementary file 1 [file animals-12-02286-s001.zip › animals-1871442-supplementary.pdf]

Air Quality, Management Practices and Calf Health in Italian Dairy Cattle Farms

Serena Bonizzi, Giulia Gislon, Milena Brasca , Stefano Morandi , Anna Sandrucci and Maddalena Zucali

Supplementary Table S1 – Questionnaire about dairy farm characteristics, herd performances and calf management practices

A - Farm description

1. In which region your farm is located?
2. Which province?
3. Which breeding system do you practice?
4. Utilized agricultural area (ha)
5. Lactating cows (number)
6. Individual daily milk production (liters per day)?
7. How many calves are born each years (number)?
8. Cows' breeds
9. Do you perform milk analysis?
10. What is the frequency of milk analysis?
11. Total number of mastitis per year
12. Average milk SCC at the herd level

B - Management practices of the calf pen

13. What types of calf pens are used?
14. How much time calves spent in single pen (days)?
15. Is there any air ventilation system in the calf pens?
16. Are there any tied calves?
17. Are there any heating lamps in calf pens?
18. Are fly treatment adopted?
19. What is the frequency of bedding renewal (days)?
20. Do you use sanitizers for bedding?
21. Have you noticed stereotypies and/or abnormal behaviour in calves?
22. What is calf pens position?

23. In what direction are the pens oriented?
24. Order of calf management in the farm work routine
25. Is there specific worker for the calves’ management?
26. Time of birth-disbudding (days)?
27. Frequency and type of calving problems
28. Do you perform navel disinfection?
29. Time birth-dam separation (hours)
30. Mortality rate from birth to 24h of life
31. Mortality rate from 24h of life to weaning
32. What are the main calves’ diseases?
33. What is the management routine at calving?
34. What are calf pens dimension?

C - Feeding

35. Time of birth-1st colostrum meal (hours)
36. What is the type of colostrum administered?
37. How do you administered colostrum?
38. Do you perform colostrum analysis?
39. Do you have a colostrum bank?
40. Milk type administered
41. How do you administer milk?
42. How many times per day do you administer milk?
43. How much milk do you administer per day?
44. Time birth-1st water offer
45. Time birth-1st starter meal
46. How much starter meal do you administer per day?
47. Time birth-1st hay offer
48. Weaning time
